# Delayed Viral Clearance Accompanied by Early Impaired Humoral and Virus-Specific T-Cell Response in Patients with Coronavirus Disease 2019 and Interstitial Lung Disease

**DOI:** 10.3390/vaccines13060655

**Published:** 2025-06-19

**Authors:** Jiaying Zhong, Juan Li, Rui Wei, Bingpeng Guo, Tingting Cui, Peiyu Huang, Zhongfang Wang, Qun Luo, Qian Han

**Affiliations:** 1 State Key Laboratory of Respiratory Disease, National Clinical Research Center for Respiratory Disease, Guangzhou Institute of Respiratory Health, The First Affiliated Hospital of Guangzhou Medical University, Guangzhou Medical University, Guangzhou 510180, China; 2 Guangzhou National Laboratory, Guangzhou 510320, China; 3 Department of Infectious Disease, Respiratory and Critical Care Medicine, Guangzhou First People’s Hospital, Guangzhou Medical University, Guangzhou 510180, China

**Keywords:** COVID-19, interstitial lung disease, SARS-CoV-2, virus-specific T-cell immune response, neutralizing antibodies

## Abstract

Objectives: Patients with interstitial lung disease (ILD) and severe acute respiratory syndrome coronavirus 2 (SARS-CoV-2) infection are at high risk of severe coronavirus disease 2019. It is unclear whether anti-viral cellular and humoral immunity is impacted in patients with ILD in the presence of immune disorders and immunosuppressive therapy. This results in poor control of viral infections following SARS-CoV-2 infection. We aimed to highlight the clinical management of patients with ILD with regard to the adjustment of anti-inflammatory therapy during SARS-CoV-2 infection. Methods: We compared viral clearance, antibody levels, and T-cell immune response between healthy controls and patients with connective tissue disease-related ILD (CTD-ILD) or interstitial pneumonia with autoimmune features (IPAF). Results: Patients with ILD exhibited a higher viral load than the control group (1.58 × 10^6^ vs. 2.37 × 10^3^ copies/mL, *p* = 0.018), as well as a significantly lower level of neutralizing antibodies against the wild-type (WT) virus (7.01 vs. 625.6, *p* < 0.0001) and Omicron BA.5 (7.19 vs. 128.4, *p* < 0.001). Similarly, a lower virus-specific T-cell (VST) immune response was observed 14 days post-symptom onset in the ILD group (CD4^+^ VSTs: 0.018 vs. 0.082, *p* = 0.005; CD8^+^ VSTs: 0.0008 vs. 0.047, *p* = 0.004). The ILD group had no other heightened inflammatory biomarkers compared with the control group. Conclusions: Our study provides novel evidence of the underlying interaction between virus clearance and host immune status and sheds light on the clinical management of patients with ILD with regard to the adjustment of anti-inflammatory therapy during SARS-CoV-2 infection.

## 1. Introduction

Coronavirus disease 2019 (COVID-19), caused by severe acute respiratory syndrome coronavirus 2 (SARS-CoV-2), significantly threatens public health. Nonetheless, the mechanisms underlying severe infection are not fully understood, and pre-existing diseases may be important risk factors [1]. Previous studies have suggested that patients with ILD have a higher risk of contracting COVID-19 and experiencing worse outcomes [2,3,4]. Connective tissue disease-related ILD (CTD-ILD) and interstitial pneumonia with autoimmune features (IPAF) account for a large percentage of ILDs, with most patients prescribed immunosuppressive therapy. It remains unclear whether anti-viral cellular and humoral immunity is impacted in patients with ILD in the presence of an immune disorder and immunosuppressive therapy. This leads to poor control of viral infections following SARS-CoV-2 infection.

We have gained a significant understanding of the natural immunity and adaptive responses to SARS-CoV-2 infection, but evidence in patients with ILD is sparse. Pertzov et al. revealed that patients with ILD prescribed anti-inflammatory therapy failed to show an adequate immune response after two doses of the BNT162b2 vaccine; the anti-spike immunoglobulin G (IgG) antibody titer was much lower than that in a healthy control group, and 48% of patients were seronegative 4–6 months after the second vaccination, indicating a delayed response and shortened duration of humoral immunity in vaccinated patients with ILD [5]. The expansion and activation of SARS-CoV-2-specific T cells also greatly contributed to virus clearance [6], especially in immunosuppressed patients. Previous studies have shown that cellular responses are not contemporaneously blunted in patients with a depressed humoral response, and a reserved T-cell response correlated with good patient survival [7]. These studies suggest that a comprehensive assessment of COVID-19 immunity may help understand virus kinetics.

Several studies have demonstrated the initiation and flare-up of autoimmune inflammatory rheumatic diseases following COVID-19 vaccination or SARS-CoV-2 infection [8,9,10]. Patients with ILD and COVID-19 infection can demonstrate hyper- or hypo-immune statuses. This poses another challenge for clinicians in maintaining a balance between controlling autoimmune diseases using immunosuppressants and stimulating host anti-viral immunity. In this study, using a small-sized ILD cohort, we compared the viral titer and humoral and cellular immunity in patients with COVID-19 and pre-existing ILD with those without underlying pulmonary disease. We attempted to explain the interaction between the virus and the host immune response in patients with ILD. We also measured several cytokines/chemokines to determine whether patients with COVID-19 and ILD had a hypercytokine response. Our study highlights the clinical management of patients with ILD and the need to adjust anti-inflammatory therapy during SARS-CoV-2 infection.

## 2. Methods

### 2.1. Study Cohort

Seventeen consecutive patients with COVID-19 and CTD-ILD/IPAF, who were admitted to the First Affiliated Hospital of Guangzhou Medical University between 5 December 2023 and 31 January 2023, were enrolled in this study. The control group comprised forty-two patients with a pre-existing Omicron BA.5 infection and no underlying pulmonary disease during the same period. COVID-19 was diagnosed according to the standard protocol established by the National Health Commission of China. Patients with COVID-19 infection were classified as severe or critically severe according to the Guidelines for the Diagnosis and Treatment of New Coronavirus Pneumonia (Fifth version, published by the National Health Commission of China) [11]. Peripheral whole blood and a nasopharyngeal swab were obtained from each patient on admission and at various time points during hospitalization. All patients authorized their clinical records to be reviewed, and the study protocol was approved by the Ethics Committee of Guangzhou Medical University (ES-2023-013-01).

### 2.2. Measurement of SARS-CoV-2 Viral Load on Nasopharyngeal Swabs

SARS-CoV-2 RNA was detected using a commercial kit (Sansure Biotech, Changsha, China) targeting the ORF1ab and N genes based on quantitative reverse transcription polymerase chain reaction (qRT-PCR) and approved by the China National Medical Products Administration (NMPA). Briefly, 45 polymerase chain reaction (PCR) cycles were performed on each specimen to quantify SARS-CoV-2 RNA using the cycle threshold (Ct) value. The Ct value was converted to the viral copy number using the SARS-CoV-2 RNA reference material (GBW(E)091132). Nucleocapsid (N) protein and open reading frame 1ab were simultaneously amplified as target genes. Samples positive for either target gene were considered positive.

### 2.3. Enzyme-Linked Immunosorbent Assay

A direct enzyme-linked immunosorbent assay of the SARS-CoV-2 nucleocapsid protein (N-protein) was performed to detect antibodies against the N-protein (anti-N IgG), following the manufacturer’s instructions (enzyme-linked immunosorbent assay [ELISA]; Darui Biotechnology, Guangzhou, China).

### 2.4. iFlash SARS-CoV-2 IgG Assay

Plasma IgG antibodies against the SARS-CoV-2 spike protein (S-protein) and N-protein (anti-S + N IgG) were identified using an iFlash-SARS-CoV-2 IgG detection kit (Shenzhen Yhlo Biotech Co., Ltd., Shenzhen, China) that is approved by the Chinese Food and Drug Administration (cFDA). The manufacturer’s cut-off value was 10 AU/mL.

### 2.5. SARS-CoV-2 Conventional Virus Neutralization Test

As previously described, plasma neutralization activity was evaluated using a cytopathic-based assay to detect cytopathogenic effects [12]. Plasma samples were tested using an initial dilution of 1:8 and then diluted in eight two-fold steps. All samples were mixed with a SARS-CoV-2 Wuhan-1 Omicron viral solution containing 100 median tissue culture infectious doses (TCID50) of the virus and incubated for 2 h at 37 °C with 5% carbon dioxide (CO_2_). The virus–plasma mixture was added to a 96-well plate containing 1.2 × 10^4^ Vero E6 cells. The plates were incubated for 4 days at 37 °C in a humidified environment with 5% CO_2_ and examined to determine the cytopathogenic effect using a Celigo Imaging Cytometer (Nexcelom Bioscience, Lawrence, MA, USA). The absence or presence of a cytopathogenic effect was determined by comparing each well with a positive control (a plasma sample with high SARS-CoV-2 neutralizing activity in infected Vero E6 cells) and a negative control (a human serum sample negative for SARS-CoV-2 based on the ELISA and neutralization assays and Vero E6 cells alone). A neutralizing antibody (nAb) titer below the detection limit was defined as a 50% inhibitory dilution titer of a half-maximal effective concentration (EC_50_) = 4.

### 2.6. Peptide Pool Design and Preparation

As previously described [13,14], the SARS-CoV-2-specific peptides were designed and synthesized; each peptide was dissolved in dimethyl sulphoxide (DMSO) at 20 µM and then pooled to form a stock. In total, 487 15-mer SARS-CoV-2 peptides (overlapping 11 amino acids) spanning the entire antigen region of the S, membrane (M), N, and envelope (E) proteins were designed using an online peptide generator (Peptide 2.0) and synthesized to over 80% purity (GL Biochem Corporation, Shanghai, China).

### 2.7. Peripheral Blood Mononuclear Cell Isolation and Ex Vivo Stimulation

The use of a virus peptide pool for PBMC stimulation has been previously established [13,14]. PBMCs (5 × 10^5^) were cultured in complete Roswell Park Memorial Institute (RPMI) medium (RPMI 1640 medium; Gibco, Grand Island, NY, USA) enriched with supplements, including 10% heat-inactivated fetal bovine serum (Biological Industries, Beit-Haemek, Israel), 100 µM minimum essential medium nonessential amino acids (Gibco), 100 U/mL of penicillin, 0.1 mg/mL of streptomycin, 2 mM L-glutamine, 25 mM 4-(2-hydroxyethyl)-1-piperazineethanesulfonic acid, 55 µM 2-mercaptoethanol, and 1 mM sodium pyruvate (Gibco). PBMCs were treated with a peptide pool containing 487 15-mer peptides (250 nM of each peptide) in 10 U/mL of recombinant interleukin (IL) 2 and 1 μM GolgiPlug (BD Biosciences, San Diego, CA, USA) for 16 h at 37 °C with 5% CO_2_.

### 2.8. Flow Cytometry

In total, 0.5–1 × 10^6^ cells were harvested from the 16 h stimulation cultures, washed, and incubated with Live/Dead Aqua V510 for 15 min on ice. The cells were then re-washed and surface-stained for 30 min on ice with the following antibodies: anti-CD3- PE-CF594 (BD Bioscience, clone UCHT1, Cat# 562280), anti-CD4-BV650 (BioLegend, San Diego, CA, USA, clone OKT4, Cat# 317435), anti-CD8-PerCPCy5.5 (BD Pharmingen™, San Jose, CA, USA, clone RPA-T8, Cat# 560662), anti-CD14-APC-cy7 (BD Pharmingen™, San Jose, CA, USA, clone MfP9, Cat# 560180), and anti-CD19 -APC-H7 (Biolegend, cloneHIB19, Cat# 302218). After fixation and permeabilization with Cytofix and Perm (BD Bioscience, San Diego, CA, USA, Cat# 554714), respectively, on ice for 20 min, intracellular staining was performed on ice for 30 min with anti-tumor necrosis factor (TNFα)-APC (BD Pharmingen™, San Jose, CA, USA, clone MAb11, Cat # 340534) and anti-IFNγ- APC-Alexa 700 (BD Pharmingen™, clone MAb11, Cat# 557995). After the final wash, the cells were resuspended in 200 μL of FACS buffer. A FACSFortessa instrument (BD Bioscience) was used to acquire data that were analyzed using FlowJo software software version 10.10.0. Supplementary Figure S1A shows the gating strategy for the analysis of antigen-specific T cells.

### 2.9. Cytokine Assays

Cytometric bead array (CBA) analysis was performed using the Human Th1/Th2 CBA kit (Jiangxi Cell Gene Biotech Co., Ltd., Nanchang, China) to detect the following cytokines: IL-1β, IL-6, IL-8, TNF-α, and interferon (IFN)-γ. Sample processing was performed following the manufacturer’s protocol, and the FCAP Array™ software (version 3.0.1) returned the data as the median fluorescence intensity (MFI) and concentration (pg/mL). Detailed methods have been reported in a previous study [15].

### 2.10. Statistical Analysis

All statistical analyses were performed using the GraphPad Prism software version 9.5.1. Statistical significance was set at *p* < 0.05. A student’s *t*-test was performed to analyze the difference in mean values between groups. The Mann–Whitney U test was performed to compare the central tendencies of the two groups (mean or median). Antibody responses were reported as geometric mean titer with a 95% confidence interval. The Kaplan–Meier was used for the survival analysis of patients with ILD and control individuals in 12 months. The Wilcoxon rank-sum test was used to compare paired continuous variables that were not normally distributed. Fractional polynomial fitting was performed on continuous variables to examine nonlinearity. Cut-off values were assigned to evaluate the significance of the *p*-value based on different statistical analysis methods. All values are presented as the mean ± standard error.

## 3. Results

### 3.1. General Condition of the Study Cohort

Between 8 December 2022 and 31 January 2023, 17 patients with ILD (11 patients with CTD-ILD and 6 with IPAF) were included in this study. The average age of those with ILD was 63 ± 8 years (nine females and eight males). According to the Chinese Expert Consensus for COVID-19 diagnosis [16], patients with ILD were categorized as having moderate illness (n = 4), severe illness (n = 10), or critically severe illness (n = 3). The proportion of patients with unfavorable outcomes within 3 months was 35.3% (n = 7). Patients’ basic sequential organ failure assessment (SOFA) score on admission, pulmonary imaging findings, and anti-inflammatory algorithms are described in Table 1.

### 3.2. Delayed Virus Clearance of SARS-CoV-2 Was Observed in Patients with ILD

**Figure 1:**
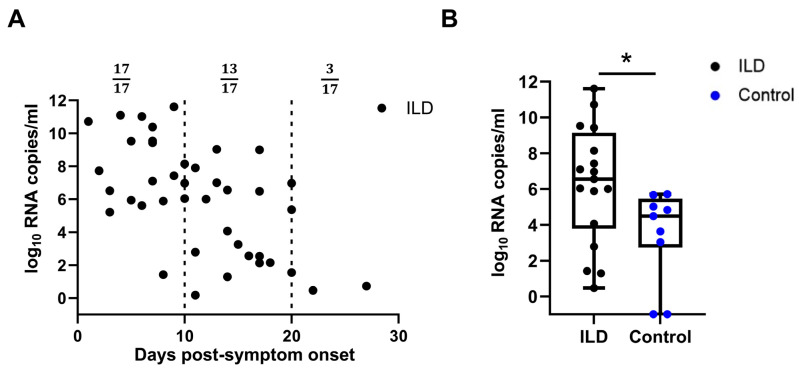
Viral loads of patients with ILD and control individuals. (**A**) Viral loads of SARS-CoV-2 at different time points post-symptom onset (PSO) in patients with pre-existing ILD (**A**). The viral load of SARS-CoV-2 in patients with ILD 10–14 days PSO was significantly higher than that in the control individuals (**B**). ILD, interstitial lung disease; SARS-CoV-2, severe acute respiratory syndrome coronavirus 2. (ns: not significant; * *p* < 0.05).

A real-time PCR analysis of the throat swabs on different days post-symptom onset (PSO) revealed more viral copies in the ILD group than in the control group. Viral load data from the ILD group indicated that 3 of 17 subjects maintained a detectable viral load 20 days post-infection (Figure 1A). The viral load was further compared between the two groups 10–14 days PSO and was found to be higher in patients with ILD than in the control individuals (1.58 × 10^6^ vs. 2.37 × 10^3^ copies/mL, *p* = 0.018), suggesting that viral clearance of SARS-CoV-2 was delayed in patients with ILD (Figure 1B). Notably, all ILD patients commenced anti-viral therapy (predominantly Paxlovid) within 24 h of SARS-CoV-2 diagnosis, per the rheumatology care protocols for this high-risk cohort. Compared with the matched controls, the ILD group demonstrated significantly earlier anti-viral initiation (median: 1.0 vs. 3.5 days post-diagnosis; *p* = 0.002) with a complete 5-day course administered to 94.1% of the ILD patients versus 62.3% of the controls (*p* = 0.003). Patients with ILD and consecutive records were selected to monitor the changes in viral load and SOFA score. A high viral load persisted as late as 3 weeks PSO, and viral rebound, corresponding to the aggravated SOFA score, was more often observed in patients with unfavorable outcomes (Supplementary Figure S2).

### 3.3. Decreased Level of Neutralizing Antibodies Against SARS-CoV-2 in Patients with ILD

**Figure 2:**
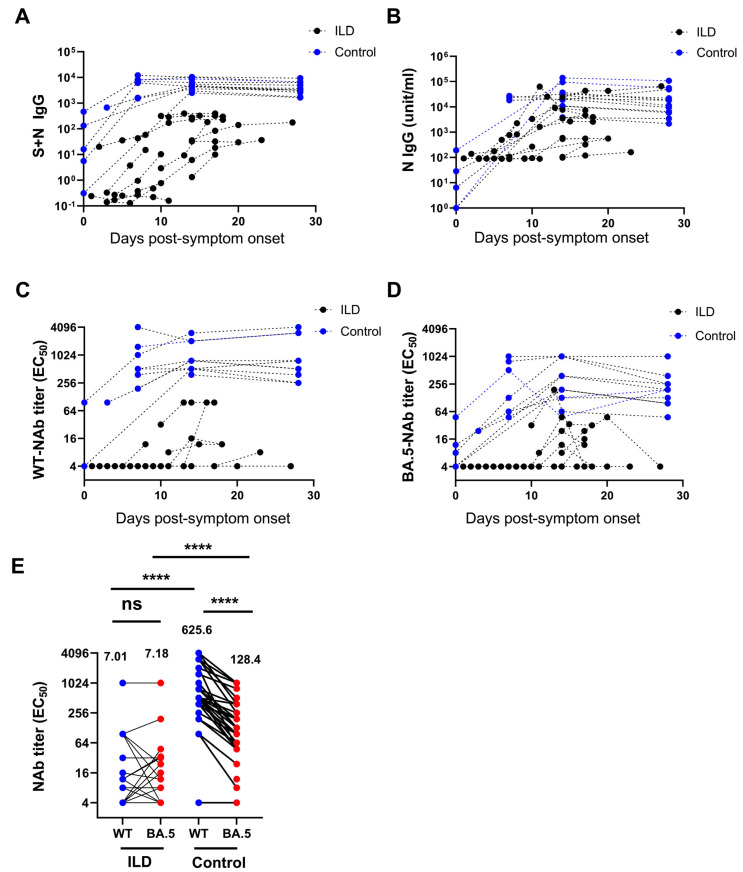
Decreased level of neutralizing antibodies against SARS-CoV-2 in patients with ILD. The level of S + N IgG (**A**) and N-IgG (**B**) against SARS-CoV-2 in patients with ILD was significantly lower than in the control group at all time points. Patients with ILD showed a significantly lower level of neutralizing antibodies against wild-type (WT) SARS-CoV-2 (**C**) and the BA.5 variant (**D**) at various time points compared with the controls. The neutralizing antibodies against the WT and BA.5 variants were comparable in patients with ILD after BA.5 infection. In contrast, the neutralizing antibodies against the WT were significantly higher than those against the BA.5 variant in control individuals (geometric means are shown) (**E**). (ns: not significant; **** *p* < 0.0001).

Compared with the control individuals, patients with ILD exhibited significantly lower S + N IgG and N-IgG levels against SARS-CoV-2 at all PSO time points (Figure 2A,B). Using a micro-neutralization assay, the ILD patients showed a significantly lower level of nAb against WT SARS-CoV-2 and the BA.5 variant at various time points following infection than the controls (Figure 2C,D). Notably, following BA.5 infection, nAbs against the WT SARS-CoV-2 and BA.5 variants were comparable in patients with ILD (7.01 vs. 7.19, *p* = 0.67, EC_50_). Nonetheless, nAbs against the wild-type were significantly higher than those against the BA.5 variant in the control individuals (625.6 vs. 128.4, *p* < 0.001, EC_50_) (Figure 2E). Similarly, patients with ILD exhibited a significantly lower nAb level against the WT virus than the control individuals (7.01 vs. 625.6, *p* < 0.0001) as well as a significantly lower level of nAb against Omicron BA.5 (7.19 vs. 128.4, *p* < 0.001). Single-patient time tendency curves showed consistently decreased antibody levels, especially in patients with unfavorable outcomes (Supplementary Figure S3).

### 3.4. Suppression of Virus-Specific T Cells Against SARS-CoV-2 in Patients with ILD

**Figure 3:**
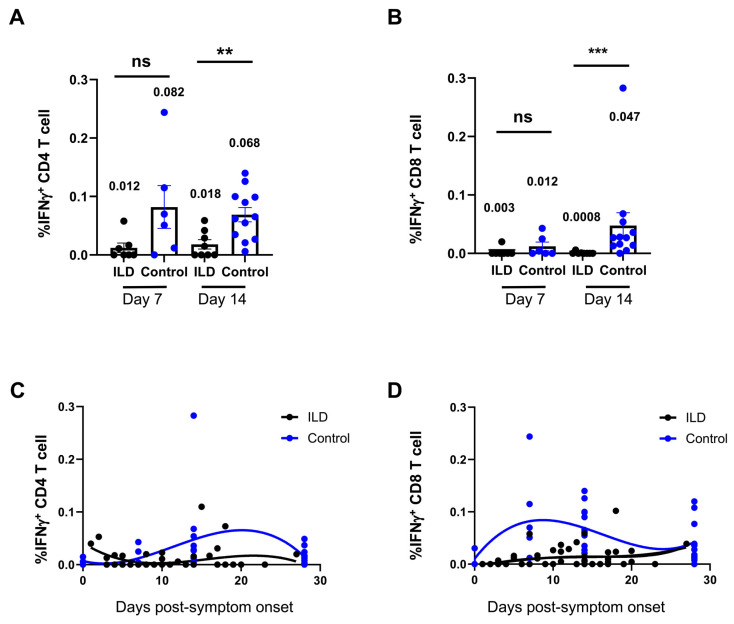
Suppression of virus-specific T cells against SARS-CoV-2 in ILD patients. (**A**) shows one patient’s representative flow cytometry plot, with the time points indicating the post-infection period. The percentages of CD4^+^ (**A**) and CD8^+^ (**B**) virus-specific T cells (VSTs) were compared between patients with ILD and control individuals 7 and 14 days PSO, respectively. Patients with ILD presented with suppressed VST profiling on days 7 and 14. In the control individuals, CD4^+^ and CD8^+^ VSTs peaked at approximately 7 and 20 days post-inoculation, respectively. The peak was not observed in patients with ILD, and the percentages of VSTs were blunted in patients with ILD 1 month PSO (**C**,**D**). Geometric means are shown (ns: not significant; ** *p* < 0.01; *** *p* < 0.001).

An ex vivo peptide stimulation method was used to assess the quantity and functionality of SARS-CoV-2 VSTs generated in patients with ILD following infection. The flow cytometry results revealed that neither CD4^+^ nor CD8^+^ VSTs initiated a response to SARS-CoV-2 in patients with ILD from 7 to 18 days PSO (Supplementary Figure S3). Compared with the control group, the percentage VSTs of CD4^+^ or CD8^+^ T cells was significantly suppressed in patients with ILD compared with the control individuals at 7 and 14 days PSO, and suppressed VST profiling was observed in patients with ILD on day 7 (CD4^+^ VSTs: ILD 0.012 vs. control 0.082, *p* = 0.06; CD8^+^ VSTs: ILD 0.003 vs. control 0.012, *p* = 0.17), persisting until day 14 (CD4^+^ VSTs: ILD 0.018 vs. control 0.082, *p* = 0.005; CD8^+^ VSTs: ILD 0.0008 vs. control 0.047, *p* = 0.004) (Figure 3A,B). The multiparameter nonlinear fitting method was applied to demonstrate the dynamic changes in VSTs in the ILD and control groups (Figure 3C,D): CD4^+^ VSTs and CD8^+^ VSTs peaked around days 7 and 20 post-inoculation, respectively, in the control individuals, whereas the peak was not observed in patients with ILD. The percentages of VSTs, including CD8^+^ and CD4^+^ cells, were blunted in patients with ILD after 1 month PSO. Similarly, a high viral load, which persisted as long as 3 weeks PSO, corresponded to the sustained suppression of VST responses (Supplementary Figure S4).

### 3.5. Inflammatory Parameters in Patients with ILD Compared with Those in the Control Group

**Figure 4:**
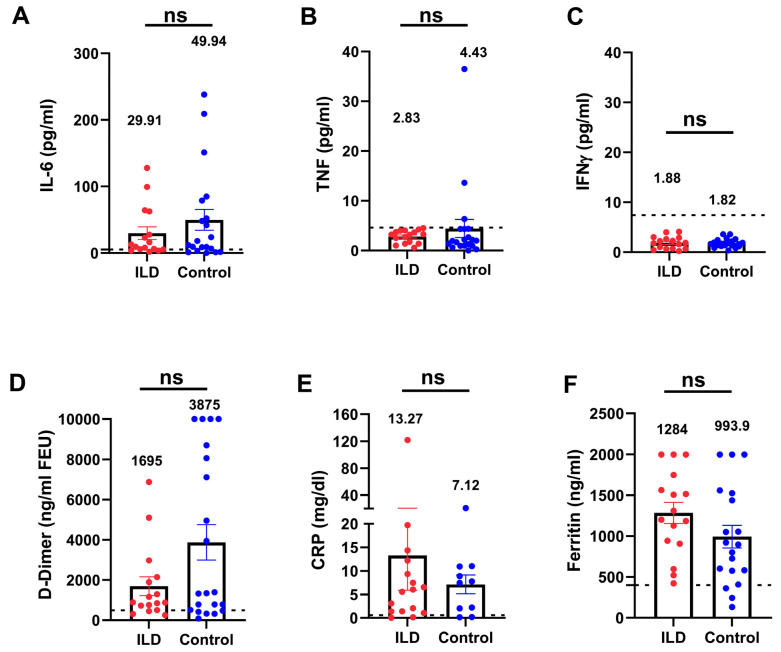
Serum inflammatory biomarkers were not elevated in patients with ILD compared with the control individuals. Patients with ILD showed no significant elevation of inflammatory cytokines (IL-6, TNF-α, and IFN-γ (**A**–**C**), D-dimer (**D**), or inflammatory biomarkers (**E**,**F**) compared with the control individuals. The dotted lines indicate the reference range. TNF-α, tumor necrosis factor-alpha; IFN-γ, interferon gamma. (ns: not significant).

**Figure 5:**
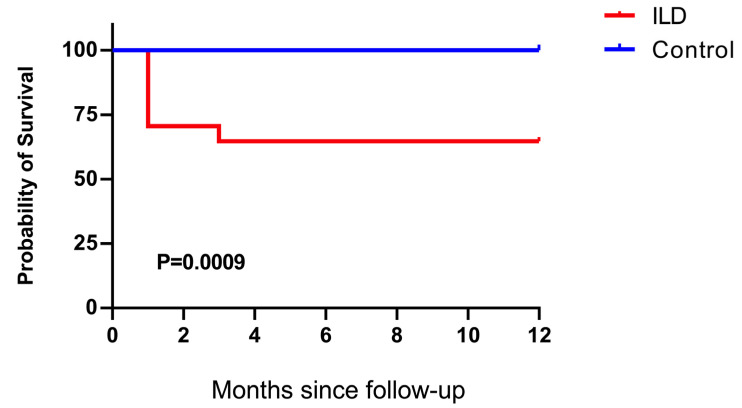
Kaplan–Meier survival analysis of patients with ILD and control individuals. The survival rate of patients with ILD was significantly lower than that of the control individuals (*p* = 0.0009).

To investigate the inflammatory status in patients with ILD and an impaired anti-viral immune response, we compared the inflammatory markers between patients with ILD and age- and severity-matched control individuals. Patients with ILD showed no significant elevation in cytokines (IL-6, TNF-α, and IFN-γ), inflammatory biomarkers, C-reactive protein, ferritin, or D-dimer (Figure 4A–F), indicating that patients with COVID-19 and ILD did not manifest additional hyperinflammation compared with the control individuals. Nevertheless, even in the absence of hyperinflammation, the rate of adverse events and the organ failure score (Figure 5) suggested a more pronounced severity of the disease trajectory among ILD patients.

## 4. Discussion

Our study demonstrated that patients with CTD-ILD/IPAF presented delayed virus clearance following infection with SARS-CoV-2, which may be attributed to the inability of B cells to generate an elevated antibody response and impaired T-cell immunity. There were no significant differences in the inflammatory biomarkers between the ILD and control groups with COVID-19. To the best of our knowledge, our study is the first to monitor virus clearance and adaptive immunity in patients with CTD-ILD/IPAF following SARS-CoV-2 infection. Nonetheless, the cohort was small.

Previous studies have demonstrated that patients with pre-existing comorbidities, such as ILDs or CTDs, are at higher risk of contracting COVID-19 and experiencing worse outcomes [17]. The patients enrolled in this study were those with CTD-ILD/IPAF whose basic treatment algorithm generally involved corticosteroids (16/17) and conventional disease-modifying rheumatic agents (DMARDs), including cyclosporin A (CsA) (3/17), tacrolimus (TAC) (7/17), mycophenolate mofetil (MMF) (5/17), and hydroxychloroquine (HCQ) (3/17). Therefore, compromised humoral and cellular immunity may have rendered these patients more prone to COVID-19 infection. The viral load was much higher in patients with ILD than in the control individuals at 10–14 days PSO, reported as the median time for viral clearance in the normal population [18]. Despite a protocol-driven immediate initiation of Paxlovid (median 0.5 days post-diagnosis) and 94.1% 5-day course completion in ILD patients—rates that are significantly superior to the controls–viral clearance remained substantially delayed compared with their immunocompetent counterparts. This implies that the profound baseline impaired immunity in patients with ILD fundamentally compromised anti-viral efficacy.

Elevated viral load in patients with ILD may be associated with suppressed immune response functions, with insufficient antibody production being a critical contributing factor [19]. Previous studies have shown higher odds of intensive care admission/mechanical ventilation in COVID-19 patients with CTD than in matched controls [20] and a compromised antibody response following SARS-CoV-2 vaccination in patients with ANCA-associated vasculitis receiving B-cell depleting therapy [21]. Critically, the observed immune impairment cannot be attributed to waning vaccine immunity or prior infection history, given our cohort’s uniform >6-month vaccine interval and primary infection status. Instead, the absence of cross-reactive antibody development following BA.5 exposure —a key immune adaptability metric—revealed profound immune inflexibility intrinsic to ILD pathophysiology. This defect likely explains both prolonged viral persistence and a high reinfection risk against emerging variants [22].

Previous studies have largely focused on the effect of antibody production on virus clearance; nonetheless, increasing evidence suggests that SARS-CoV-2-specific T-cell immunity is crucial to an adequate response to infection and vaccination [23,24]. Our results showed suppressed CD4^+^ and CD8^+^ VSTs in patients with ILD on days 7 and 14 after PSO. These results are consistent with those of studies in patients who underwent organ transplantation and who demonstrated impaired humoral and cellular immunity following SARS-CoV-2 prime-boost vaccination (BNT162b2) [25] or a delayed peak of VSTs that appeared 29 days PSO (unpublished data). Another study in patients with hematological cancer and COVID-19 revealed that 77% had detectable SARS-CoV-2-specific T-cell responses despite impaired humoral immunity, including those treated with anti-CD20 therapy, and those with several CD8^+^ T cells had improved survival [26]. We further found a delayed peak in CD4^+^ VSTs and decreased levels of CD4^+^ and CD8^+^ VSTs in patients with ILD, suggesting a suppressed T-cell response during the first month. Therefore, an immunosuppressed profile was demonstrated in patients with CTD-ILD/IPAF after SARS-CoV-2 infection. Notably, all conventional DMARDS (including CsA, Tac, MMF, and HCQ) were de-escalated following a positive SARS-CoV-2 diagnosis; nonetheless, cumulative exposure to immunosuppressive therapy and/or autoimmune diseases may be responsible for the blunted immunosuppressive response.

Immunosuppressive therapy is recommended for severe COVID-19 to counter hyperinflammation-mediated organ damage [27]. Although comparable inflammatory profiles were observed in ILD patients and the controls, initiating immunosuppression in COVID-19-ILD cases presents a critical paradox: while potentially controlling cytokine storms, it risks impairing viral clearance and accelerating disease progression [28]. Evidence of the effect of antecedent and continuous pharmacological immunosuppression on the prognosis of patients with COVID-19 and rheumatic diseases is controversial [29,30,31,32,33]. The effect of the treatment algorithm highly depends on the type of anti-inflammatory drugs, activity of underlying CTDs, disease phase of COVID-19, and related comorbidities. Coupled with our finding of markedly worse survival in ILD patients despite similar initial inflammation (attributable to blunted anti-viral immunity), we propose the following clinical framework: (1) Anti-viral agents (e.g., nirmatrelvir/ritonavir) must be prioritized during active infection; (2) immunosuppression should generally be deferred until viral clearance is confirmed (antigen/PCR-negative), typically ≥7 days post-symptom onset; (3) for patients with critical rheumatic disease flares necessitating immunosuppression during infection, minimal effective doses of corticosteroids + concurrent anti-virals should be prescribed and nonessential immunomodulators avoided. Serial viral load monitoring is mandatory to prevent viral rebound.

This study has some limitations. First, the small sample size was particularly reflected in limited power for day-7 analyses (post-hoc power = 0.40), and restriction to hospitalized patients prevents the generalizability of results. Second, owing to the limited PBMC volume and small cohort size, we did not perform dedicated B-cell phenotyping; therefore, correlations between antibody titers, B-cell subsets, and the influence of immunosuppression remain to be explored in future, larger studies employing multiparameter flow cytometry. Future studies should also incorporate IL-18 profiling, given its central regulatory role in T/NK cell activation and cytokine storm pathogenesis, alongside IFN-γ/TNF-α analyses to distinguish specific immune dysregulation signatures in SARS-CoV-2-infected ILD patients. Third, longitudinal monitoring beyond discharge and extended follow-ups in multicenter cohorts are needed to fully characterize post-acute viral/immune kinetics and optimize prophylactic/therapeutic strategies.

## 5. Conclusions

We demonstrated prolonged viral clearance in patients with ILD and rheumatic or autoimmune diseases following SARS-CoV-2 infection, coinciding with depressed virus-specific humoral and cellular immunity. Comparable inflammation profiles were observed between the ILD and control groups. Considering the shift in SARS-CoV-2 infection towards a seasonal pattern, our findings provide a rationale for enhanced immune monitoring in at-risk CTD-ILD/IPAF populations and offer implications relevant to the management of broader viral infections beyond COVID-19 in the post-pandemic era.

## Figures and Tables

**Table 1:** Characteristics of the study’s participants.

Case Number	Sex	Age	ILD Classification	Inactivated Vaccination History *	Severity of COVID-19	SOFA on Admission	Lung Infiltrates (%)		Anti-Inflammatory		3-Month Outcome
							Acute	Basic	MP (mg/day)	Immuno-Suppressant	
C1	F	67	IPAF	No	Severe	2	17	3	4	CsA	Survive
C2	F	59	CTD-ILD	No	Severe	1	26	32	4	CsA	Dead
C3	M	65	CTD-ILD	No	Critically severe	3	44	17	4	CsA	Dead
C4	M	68	IPAF	One dose	Severe	0	6	7	4	—	Survive
C5	F	55	CTD-ILD	No	Moderate	0	10	0	4	Tac, HCQ	Survive
C6	F	65	CTD-ILD	No	Severe	0	18	12	8	Tac	Survive
C7	M	63	CTD-ILD	No	Severe	2	2	9	16	Tac, HCQ	Survive
C8	M	43	IPAF	Three doses	Severe	0	24	13	12	MMF	Lung transplantation
C9	F	55	IPAF	Three doses	Moderate	0	12	2	—	Tac	Survive
C10	F	67	CTD-ILD	Three doses	Severe	1	36	3	12	—	Survive
C11	M	59	CTD-ILD	Three doses	Moderate	2	28	18	—	MMF, Tac, HCQ	Survive
C12	M	72	IPAF	Three doses	Severe	0	25	34	8	Tac	Survive
C13	M	62	IPAF	Three doses	Critically severe	3	32	40	—	MMF	Dead
C14	F	59	CTD-ILD	Three doses	Severe	3	21	1	4	MMF	Dead
C15	M	59	CTD-ILD	Three doses	Severe	1	17	15	8	MMF	Survive
C16	F	83	CTD-ILD	No	Critically severe	4	58	0	—	—	Dead
C17	F	70	CTD-ILD	No	Moderate	1	—	—	8	Tac	Survive

F: female; M: male; IPAF: interstitial pneumonia with autoimmune features; CTD-ILD: connective tissue disease-related interstitial lung disease; CsA: cyclosporin; Tac: tacrolimus; HCQ: hydroxychloroquine; MMF: mycophenolate; MP: methylprednisolone. * Inactivated vaccine administration protocol: Two intramuscular doses (0.5 mL/dose) administered 21–28 days apart, with a third booster dose ≥6 months later.

## Data Availability

All data relevant to the study are included in the article or uploaded as Supplementary Information.

## Supplementary Materials

The following supporting information can be downloaded at: https://www.mdpi.com/article/10.3390/vaccines13060655/s1, Figure S1. Gating strategy for analysis of virus-specific T cells; Figure S2. Single patient time tendency curves describing viral loads and SOFA scores; Figure S3. Single patient time tendency curves describing—Nab titre; Figure S4. Single patient time tendency curves describing T-cell immunity.
