# Supplementary material for: Delayed Viral Clearance Accompanied by Early Impaired Humoral and Virus-Specific T-Cell Response in Patients with Coronavirus Disease 2019 and Interstitial Lung Disease
Source: Vaccines (Basel). 2025 Jun 19;13(6):655. doi: 10.3390/vaccines13060655 (PMC12197558; doi:10.3390/vaccines13060655)
Supplement: Supplementary file 1 [file vaccines-13-00655-s001.zip › vaccines-3606810-supplementary.pdf]

Supplementary figure legends

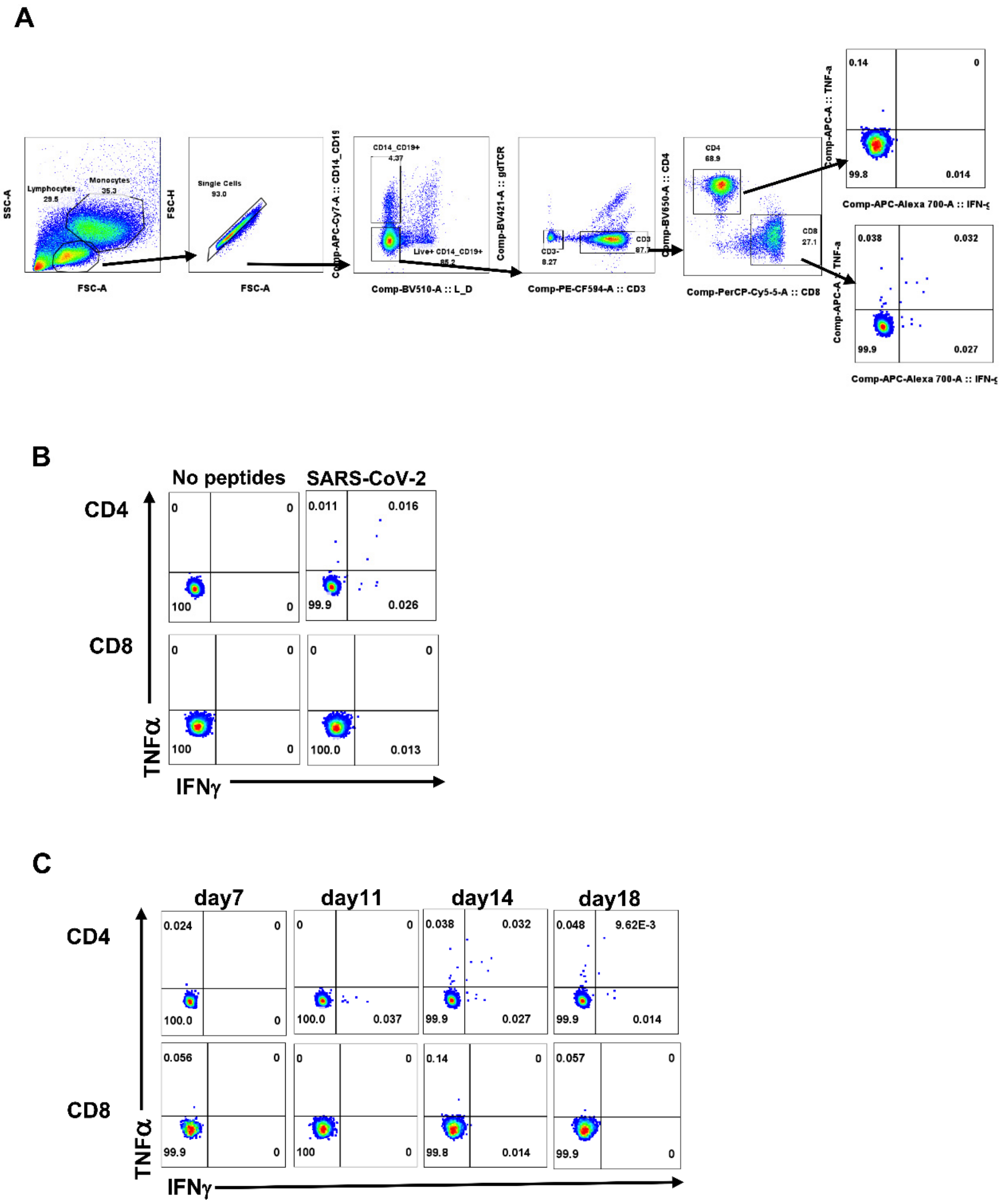

Supplement FigureS1

Supplementary Figure S1. Gating strategy for analysis of virus-specific T cells

A PBMCs were stimulated overnight with SARS-CoV-2 peptides and then were surface and intracellularly immuno-stained. Live CD3 were gated from singlets derived from lymphocytes, and based on live CD3, CD4 and CD8 were further gated on IFN-γ and

TNF-α. B presentative flow cytometry dot plots, showing the patient's virus-specific T cell data, and setting up both the peptide stimulation group（SARS-CoV-2） and the NC group （no peptides）. C shows a representative dot plot of virus-specific T cells in a patient at each time point.PBMC, peripheral blood mononuclear cells; IFN-γ, interferon-gamma

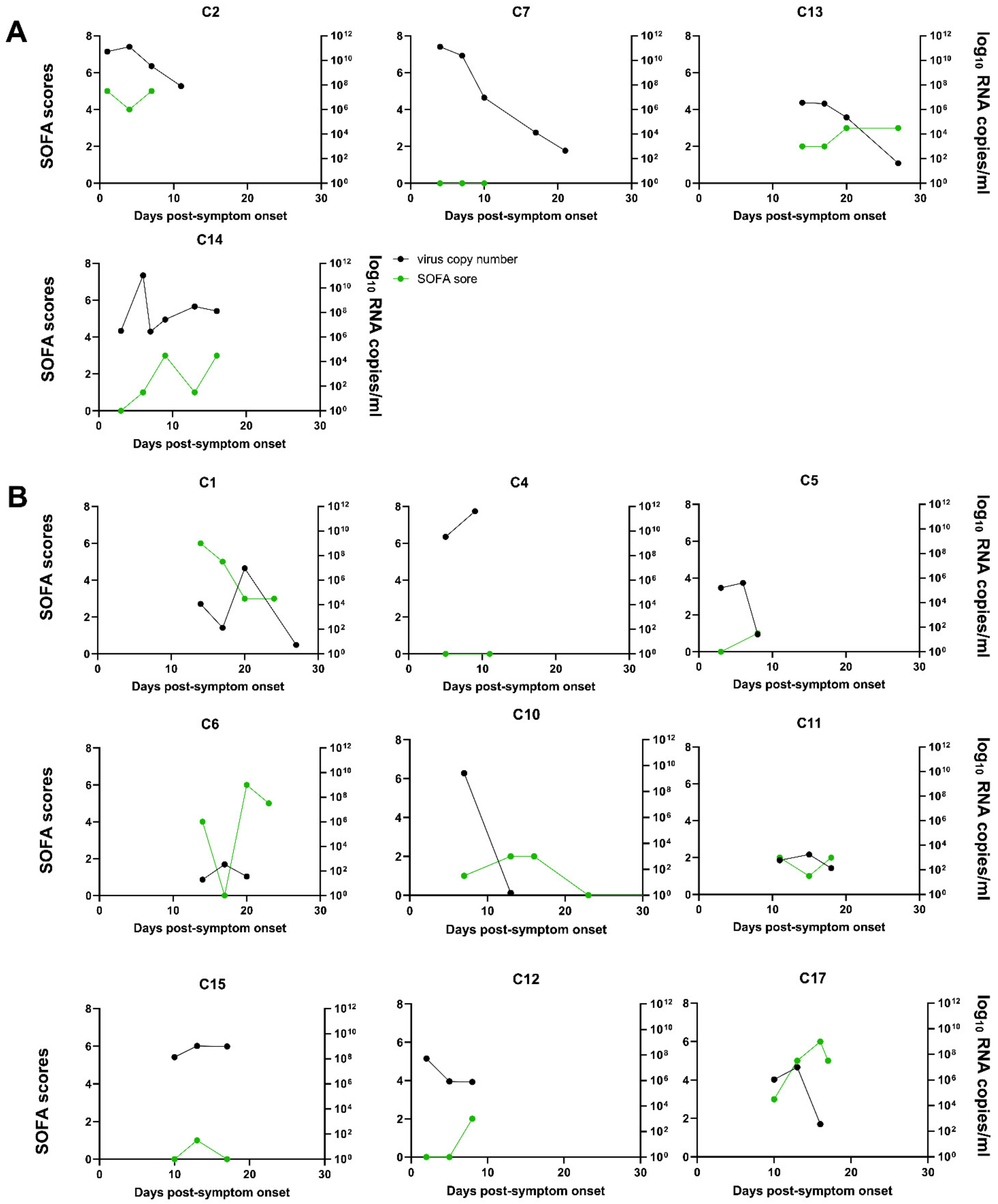

Supplement FigureS2

Supplementary Figure S2. Single patient time tendency curves describing viral loads and SOFA scores

Patients with ILD with consecutive records were selected to monitor the changes in viral loads and SOFA scores. The high levels of viral load lasted till 3 weeks PSO, and the viral rebound, in correspondence with the aggravation of SOFA scores, were more often observed in patients with unfavourable outcomes.

SOFA, sequential organ failure assessment; PSO, post-symptom onset

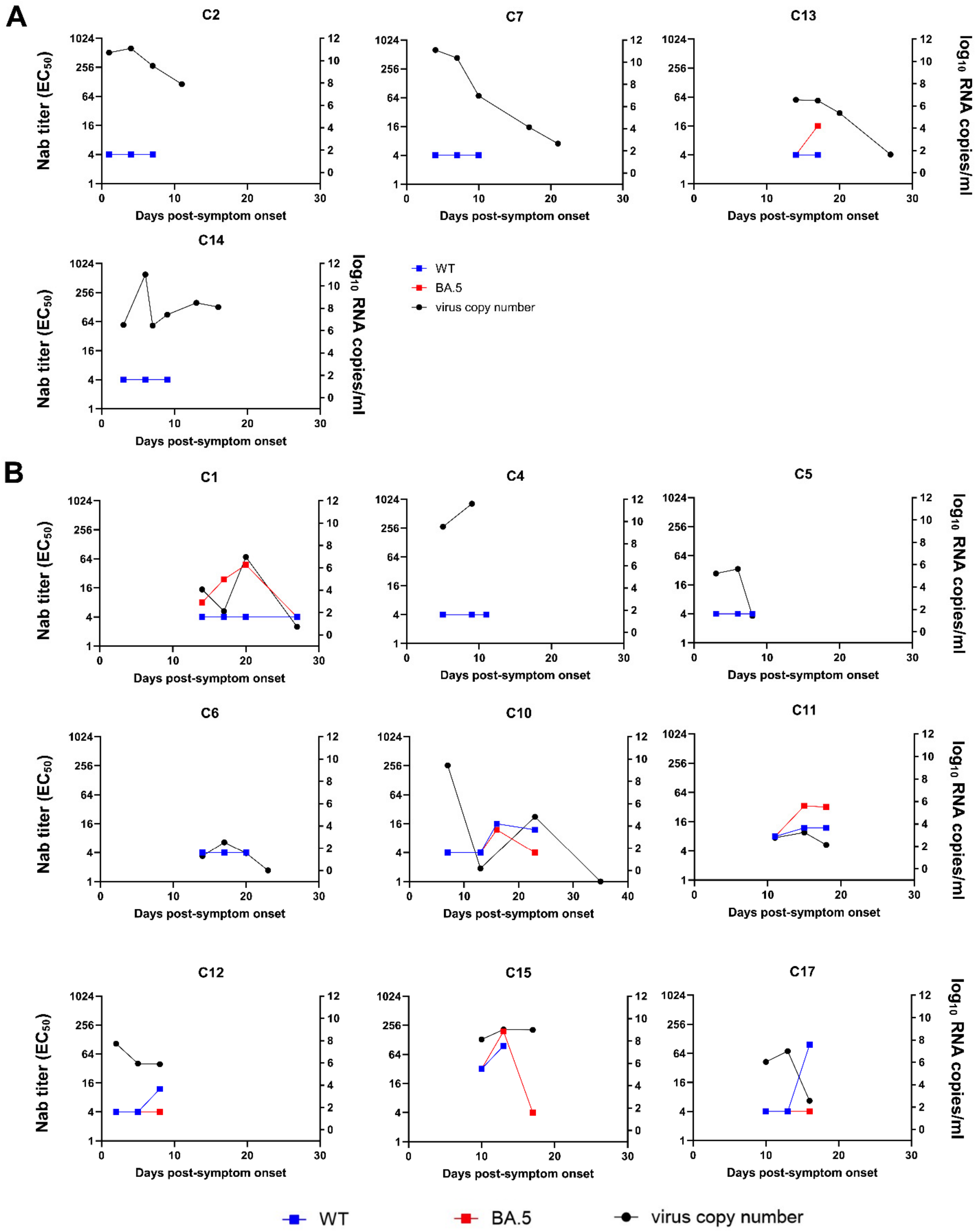

Supplement FigureS3

Supplementary Figure S3. Single patient time tendency curves describing - Nab titre

Patients with ILD with consecutive records were selected to monitor changes in nAb titres and viral loads, indicating a consistent depressed B-cell response, especially in patients with unfavourable outcomes.

nAb, Neutralizing antibodies

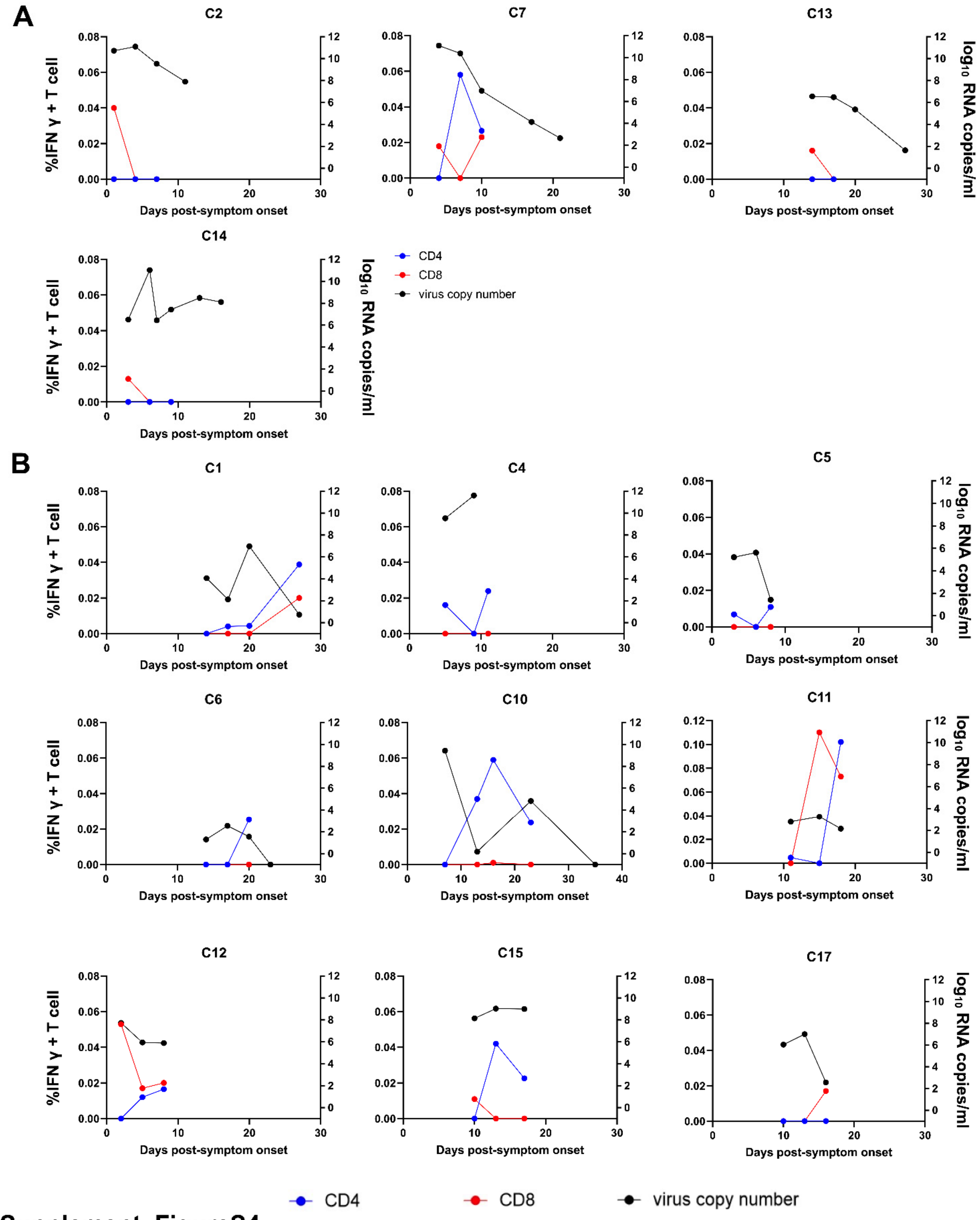

Supplement FigureS4

Supplementary Figure S4. Single patient time tendency curves describing T-cell immunity

Patients with ILD with consecutive records were selected to monitor the changes in B-cell immunity and viral loads, and the high levels of viral load, which lasted till 3 weeks PSO, corresponded with the sustained suppression of virus-specific T-cell responses.
